# [Corrigendum] Pharmacokinetics and antitumor efficacy of DSPE-PEG2000 polymeric liposomes loaded with quercetin and temozolomide: Analysis of their effectiveness in enhancing the chemosensitization of drug-resistant glioma cells

**DOI:** 10.3892/ijmm.2025.5530

**Published:** 2025-04-08

**Authors:** Jun Hu, Junjie Wang, Gang Wang, Zhongjun Yao, Xiaoqian Dang

Int J Mol Med 37: 690-702, 2016; DOI: 10.3892/ijmm.2016.2458

Following the publication of the above article, an interested reader drew to the Editor's attention that data in certain of the figures appeared to be strikingly similar to that included in three other articles that had been contributed to other publications by the same research group. After having investigated the matter in the Editorial Office in conjunction with the authors, it transpired that certain of the flow cytometric (FCM) data featured in Fig. 4C on p. 697 had been placed erroneously in the published version of this figure, and were duplicates of data featured correctly/as intended in another one of this group's papers. Since the cell experiments in both studies were conducted simultaneously, the subsequent cell growth pictures were also taken at the same time, and the data were inadvertently mislabelled. Upon reviewing their data, the authors realized that the images of the QUE-NLs (50 μM), QUE-NLs (100 μM) and QUE-NLs (200 μM) administration groups in Fig. 4C were the FCM plots that had been featured incorrectly in this figure (as indicated by the green labels in the corrected version of Fig. 4, which is shown on the next page). Furthermore, the cell growth images shown in Fig. 4B for the 'DMSO' and 'Control-NLs' experiments had also been assembled incorrectly in this figure; the correct images for the 'DMSO' and 'Control-NLs' data panels are also highlighted by the green labels in the corrected version of Fig. 4 on the next page.

The authors can confirm that the errors made in assembling this figure did not have any significant impact on either the results or the conclusions reported in this study, and all the authors agree with the publication of this Corrigendum. The authors are grateful to the Editor of *International Journal of Molecular Medicine* for allowing them the opportunity to publish this; furthermore, they apologize to the readership of the Journal for any inconvenience caused.

## Figures and Tables

**Figure 4:**
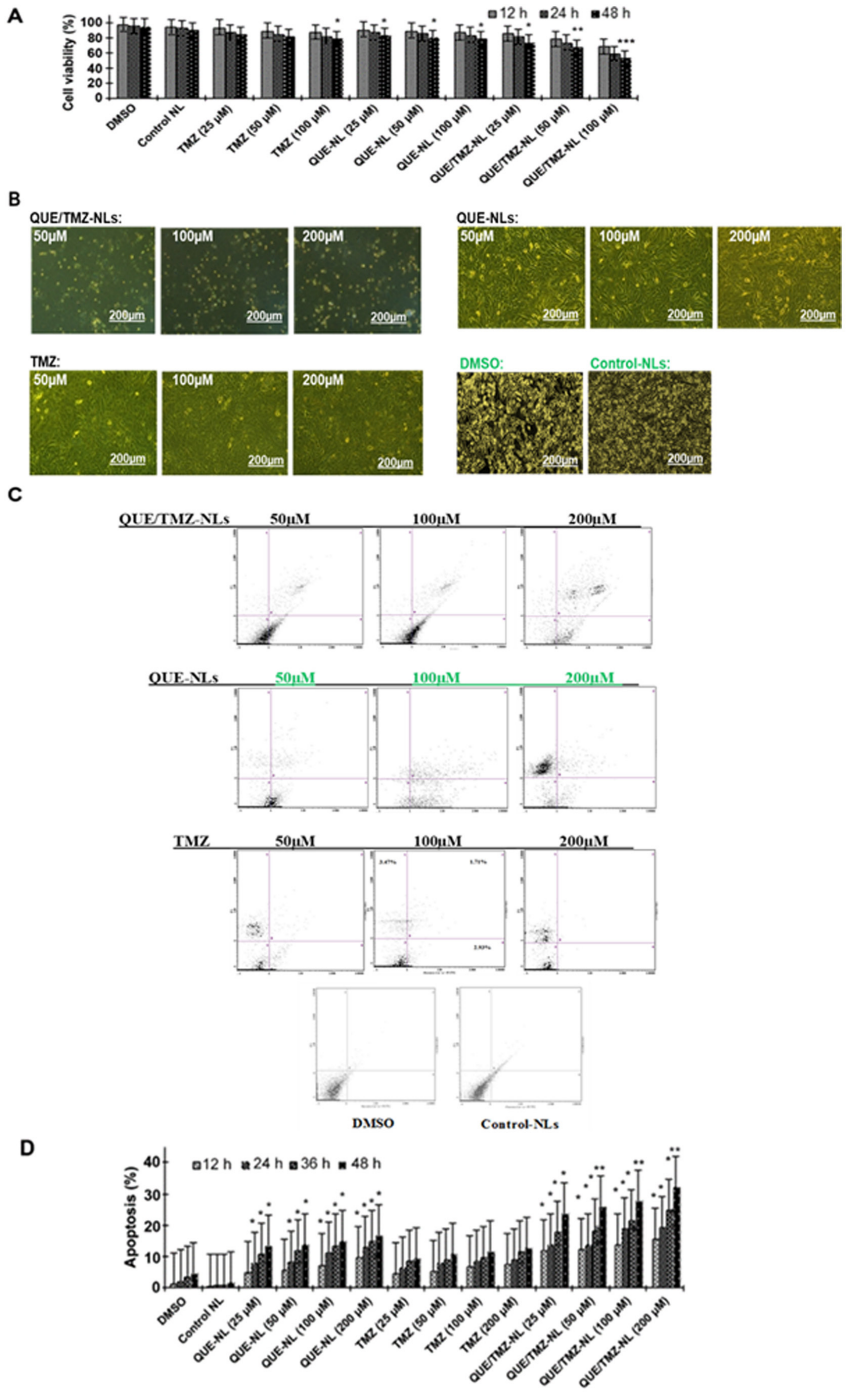
Quercetin and temozolomide-loaded nanoliposomes (QUE/TMZ-NLs) exert cytotoxic effects on the U87 glioma cells. (A) Treatment with QUE/TMZ-NL decreased U87 cell viability. Each point is the mean ± SD of 3 experiments. (B) Cell morphology was examined using a phase contrast microscope. Representative images of the indicated treatments are shown. (C and D) QUE/TMZ-NLs induced the apoptosis of the U87 cells, as measured by Annexin V-FITC/PI staining and flow cytometry. The cells were cultured with the indicated concentrations of QUE/TMZ-NLs, free QUE or TMZ for 12, 24, 36 and 48 h. Each point is the mean ± SD of 3 experiments. ^*^p<0.05, ^**^p<0.01 and ^***^p<0.001 compared with the control. QUE, quercetin; TMZ, temozolomide; NLs, nanoliposomes; PI, propidium iodide.

